# Availability and Use of Therapeutic Interchange Policies in Managing Antimicrobial Shortages among South African Public Sector Hospitals; Findings and Implications

**DOI:** 10.3390/antibiotics9010004

**Published:** 2019-12-20

**Authors:** Audrey K. Chigome, Moliehi Matlala, Brian Godman, Johanna C. Meyer

**Affiliations:** 1 Division of Public Health Pharmacy and Management, School of Pharmacy, Sefako Makgatho Health Sciences University, Ga-Rankuwa, Pretoria 0208, South Africa; audreychigome@gmail.com (A.K.C.); hannelie.meyer@smu.ac.za (J.C.M.); 2 Strathclyde Institute of Pharmacy and Biomedical Sciences, Strathclyde University, Glasgow G4 ORE, UK; 3 Department of Laboratory Medicine, Division of Clinical Pharmacology, Karolinska Institute, Karolinska University Hospital Huddinge, SE-141 86 Stockholm, Sweden; 4 Health Economics Centre, Liverpool University Management School, Chatham Street, Liverpool L69 7ZH, UK

**Keywords:** antimicrobial shortages, antimicrobial stewardship, therapeutic interchange, pharmacists, public sector hospitals, South Africa

## Abstract

Background: Therapeutic interchange policies in hospitals are useful in dealing with antimicrobial shortages and minimising resistance rates. The extent of antimicrobial shortages and availability of therapeutic interchange policies is unknown among public sector hospitals in South Africa. This study aimed to ascertain the extent of and rationale for dealing with antimicrobial shortages, describe policies or guidelines available, and the role of pharmacists in the process. Methods: A quantitative and descriptive study was conducted with a target population of 403 public sector hospitals. Data were collected from hospital pharmacists using an electronic questionnaire via SurveyMonkey^TM^. Results: The response rate was 33.5% and most (83.3%) hospitals had experienced shortages in the previous six months. Antimicrobials commonly reported as out of stock included cloxacillin (54.3%), benzathine benzylpenicillin (54.2%), and erythromycin (39.6%). Reasons for shortages included pharmaceutical companies with supply constraints (85.3%) and an inefficient supply system. Only 42.4% had therapeutic interchange policies, and 88.9% contacted the prescriber, when present, for substitution. Conclusions: Antimicrobial shortages are prevalent in South African public sector hospitals with the most affected being penicillins and cephalosporins. Therapeutic interchange policies are not available at most hospitals. Effective strategies are required to improve communication between pharmacists and prescribers to ensure that safe, appropriate, and therapeutically equivalent alternatives are available.

## 1. Background

Sub-Saharan African countries including South Africa have the highest burden of infectious diseases worldwide, enhanced by high prevalence rates of HIV, malaria, and tuberculosis [1,2,3], with antimicrobials crucial to reducing the burden of communicable diseases [4]. On one hand, there are concerns regarding the inappropriate use of antibiotics leading to increasing rates of antimicrobial resistance (AMR) limiting therapeutic options, which have resulted in the introduction of antimicrobial stewardship programmes (AMSPs) across countries acknowledging though the challenges [5,6,7,8]. However, shortages of antimicrobials are also a challenge in Africa [9,10,11], potentially resulting in the use of antibiotics that place patients at a greater risk of *Clostridium difficile* infections as well as increased AMR [12,13].

The medicine shortage situation in South Africa is also a concern receiving media attention [14,15,16]. As a result, national monitoring programmes have been put in place in South Africa with the support of the National Department of Health (NDoH). These include improved supply management programmes as well as the ‘Stop Stockouts’ initiative, which is a consortium of civil society organisations [17,18]. Despite these initiatives, shortages are still being experienced in some provinces in South Africa [16,18,19]. Shortages of antimicrobials are regarded as a public health emergency due to the necessity to expedite treatment in the case of an infection and because escalating AMR rates limit therapeutic options for many pathogens [20,21]. 

Where shortages of antimicrobials exist, therapeutic interchange policies can potentially help ensure that substitution is not haphazard or inappropriate [22]. Pharmacists can play a crucial role in the development and implementation of therapeutic interchange policies in hospitals combined with other key stakeholder groups, building on activities within Pharmacy and Therapeutic Committees (PTCs) as well as AMSPs [8,23,24]. Whilst the effective functioning of PTCs is improving in South Africa and AMSPs are evolving [23,24], there are concerns regarding the extent of therapeutic interchange policies among public sector hospitals in South Africa, especially regarding antimicrobials. The NDoH published a policy in 2017 to provide guidance for the placement of medicines in classes to support therapeutic interchange programmes where pertinent [25]. However, the policy only provides guidelines on required procedures when switching a patient from one medicine to another within the same therapeutic class, but not on therapeutic interchanges where this is not possible such as antimicrobials. As a result, there is a need to build on this initiative.

Consequently, this study sought to identify and describe the current therapeutic interchange policies in the event of drug shortages among public sector hospitals in South Africa following the NDoH initiative. Antimicrobials were chosen in light of the extent of infectious diseases in sub-Saharan Africa, with public sector hospitals chosen as they offer health services to approximately 80% of the population in South Africa [17]. The findings can be used to guide future policies in South Africa as well as possibly wider.

## 2. Materials and Methods

We used a descriptive survey design and a quantitative research approach. All public sector hospitals including district, regional, and tertiary/academic hospitals (*n* = 403) in the nine provinces of South Africa were targeted for participation. One pharmacist from each hospital, in most instances the ‘Drug Controller’, ‘Procurement Pharmacist’, or ‘Pharmacy Manager’, were purposively selected to complete the questionnaire. In hospitals where none of these were available, a qualified pharmacist or community service pharmacist was requested to participate.

Data were collected from March to July 2018. The survey was conducted using a 23 item electronic questionnaire administered via SurveyMonkey™. A questionnaire link was sent via email or fax to pharmacists who agreed to take part with anonymous completion. The questionnaire was compiled, based on the published literature [11,22,26,27,28,29], after which an expert review followed. It was divided into three sections: demographics, antimicrobial shortages, and therapeutic interchange. The questionnaire also included information on current PTC- and AMSP activities in the hospitals.

Data were exported from SurveyMonkey™ to Microsoft Office Excel™ and cleaned prior to analysis using the Statistical Package for the Social Sciences (SPSS) version 25. Open-ended responses were typed into MS Excel™, and relevant categories were created to allow for the counting of responses. Descriptive statistics were used to summarise data using means with standard deviation (SD) and frequency counts with percentages. Antibiotics were classified by Anatomical Therapeutic Chemical (ATC) class [30]. All analyses were conducted at a 95% confidence interval.

Ethical clearance for the study was granted by the Sefako Makgatho University Research Ethics Committee (SMUREC/P/269/2017:PG), and permission was obtained from the NDoH.

## 3. Results

### 3.1. Response Rate and Demographic Details

Of the 403 hospitals, approval to conduct the study was received for 346 hospitals (Table 1). Of these 346 hospitals, 81 facilities could not be reached using email addresses and 11 declined to participate. A total of 85 responses were finally received, giving a response rate of 33.5% (Table 1). 

More than half of the respondents were female (63.5%), with a mean age of 38 years (SD: 9.8) (Table 2). The mean number of years in practice was 9.3 years (SD: 7.42), with 62.2% having less than 10-years of experience. Almost half (48.2%) were pharmacy managers, with most working at district hospitals (Table 2).

### 3.2. Antimicrobial Shortages

The majority of respondents (83.3%) indicated they had experienced antimicrobial shortages at their institutions in the previous six months. Most shortages were for antibiotics, particularly penicillins, with a limited number in the antiviral (acyclovir) and antifungal (amphotericin B, clotrimazole) classes (Table 3). Most of the shortages exceeded 40 days.

### 3.3. Reasons for Antimicrobial Shortages

The majority of respondents (85.3%) stated that supply problems with pharmaceutical companies were the main contributor to shortages (Table 4). The impact included dispensing later generation (84.4%) and more expensive alternatives (64.9%). The procedures for reporting medicine shortages included sending weekly reports to the central/provincial office or the district pharmacist (Table 4).

A considerable number of facilities had active PTCs (86.9%) and Antimicrobial Stewardship Committees (AMSCs) (70.2%). Most respondents were members of PTCs (77.4%) and AMSCs (51.2%). 

### 3.4. Therapeutic Interchange Policies

Less than half (42.4%) of respondents stated they had therapeutic interchange policies in place in their hospitals, of whom 30.2% reported that these were documented (Table 5). Pre-consultation with the prescriber before substitution was a requirement in 95.2% of the facilities. The extent of policies, their development, actions taken by the pharmacist, and substitution procedures followed in the absence of policies, are documented in Table 5.

### 3.5. Role of the Pharmacist in Therapeutic Interchange

More than a third of the respondents (37.1%) said the pharmacists should communicate with prescribers regarding suitable therapeutic options when there are shortages as well as facilitate the development of therapeutic interchange policies (22.9%) to improve subsequent care (Table 6).

## 4. Discussion

We believe this is the first comprehensive study among public sector hospitals in South Africa to review the current situation regarding therapeutic interchange policies following NDoH guidance [25]. The response rate of 33.5%, despite sending up to 12 reminders, which was similar to one study conducted in the US (40%) [31]; however, it was higher than studies in Europe and another study in the US with response rates of 22% and 13%, respectively [29,32]. Pharmacy managers provided the highest number of responses (Table 2), similar to a study conducted in the US [33]. 

The majority of participating hospitals had experienced antimicrobial shortages in the preceding six months, similar to studies conducted in Australia, Europe, and the US [32,34]. Shortages were either reported to the pharmacy manager, the PTC, hospital management, or the provincial office by the pharmacists, similar to the findings in a US survey [31]. It is important that shortages are reported to the relevant authorities and immediately communicated to prescribers so that pertinent strategies can be urgently identified and implemented.

The majority of shortages reported were for penicillins, followed by cephalosporins (Table 3), similar to findings in the US, Europe, and Australia [10,11,32,34]. Most of the shortages lasted more than 40 days. This is a concern, although shortages between one week and over a year have been seen in Europe and the US [11,32]. Pharmaceutical companies with supply or capacity problems, an inefficient supply system, poor stock control and financial resources were the main reasons for shortages (Table 4). However, new initiatives have recently been introduced in South Africa to help improve stock control, and we will be investigating their impact in future studies [17].

Most pharmacists reported that they had resorted to dispensing later generation antimicrobials or more expensive alternatives when faced with shortages, similar to studies in Europe and Australia [32,34]. This though may potentially hinder AMS efforts as shortages of one antimicrobial can result in shortages of others used for substitution, potentially negatively impacting on patient outcomes [35]. 

Encouragingly, the majority of the respondents said that their hospitals had active PTCs and AMSCs. This is welcomed, especially as one of the aims and objectives of the South African National Drug Policy is to establish and strengthen PTCs in all hospitals [23,24,36]. However, of concern was that less than half of the respondents (42.4%) stated their hospitals had therapeutic interchange policies. Of these, only nine had policies or substitution guidelines from their institutional PTCs. This is consistent though with a recent survey conducted among public sector hospitals in South Africa that found that the development and implementation of guidelines other than the formulary is not a primary function of hospital PTCs [23]. In addition, only 33% of participants referred to the NDoH notice to develop their therapeutic interchange policies. This needs to be addressed as it is difficult to adhere to and revise guidelines that are not documented and actively communicated. 

Half of the respondents stated that therapeutic interchange policies were the responsibility of PTCs, with PTCs generally considered the most ideal setting for such programmes, which is encouraging as they can ensure that any guidelines are based on scientific evidence and any national Essential Medicine List [17,37,38,39]. Several actions were reported as part of the pharmacist’s practice during substitution (Table 5) including communication with other health professionals and providing information on available alternatives. This is also important as decisions should be made in collaboration with all key stakeholders [35]. Encouragingly, very few respondents stated they substituted without consulting the prescriber, with 92.5% stating that pre-consultation with the prescriber was a requirement before substitution in their hospital. This is important for building trust among teams. The opposite was observed in the US where 67% of institutions performed automatic substitutions preapproved by PTCs [31]. More than a quarter of respondents in our study stated that they sent the patient back to the prescriber when a prescribed medicine was unavailable, implying that shortages were not always communicated in a timely manner. This needs to be addressed as it is important that pharmacists actively communicate with prescribers and help jointly develop therapeutic interchange policies to optimize patient care and minimize patient inconvenience [22,40].

Encouragingly, more than half of the respondents undertook keeping a record of the interchange. Additionally, it was encouraging that respondents emphasised the importance of communication with prescribers and active participation in research related to substitution decisions as well as facilitation of therapeutic interchange policy development (Table 6) in going forward. We will be building on this in future studies. Finally, some pharmacists have highlighted the importance of effective communication channels between the National/Provincial Department of Health and health facilities. This is consistent with the WHO’s recommendation to scale up systems at a national level that collect and monitor data on medicines availability for better evidence-based policy making [41].

The study had several limitations. We acknowledge there was a relatively low response rate, more than half of the respondents had less than 10-years of experience, and the responses were self-reported. It is also likely that not all shortages were accounted for due to recall bias. Furthermore, there appeared to be differences in the interpretation of therapeutic interchange policies among the pharmacists taking part. Despite these limitations, we believe that the study was robust, providing an insight into the current status of antimicrobial shortages and interchange policies among public sector hospitals in South Africa. 

## 5. Conclusions and Recommendations

From our findings, it is evident that most South African public sector hospitals experience antimicrobial shortages, particularly for penicillins and cephalosporins. These shortages may last in excess of 40 days, which can affect service delivery, AMS efforts, and patient outcomes. Of concern is that the practice of therapeutic interchange is currently not that common among public sector hospitals in South Africa and not entirely understood by pharmacists working in these hospitals. Efforts should be made to train PTC members on how to develop evidence-based policies to manage medicine shortages in their institutions, with pharmacists playing a key role based on their training. There should also be increased communication with depots and ordering early, considering depot lead times.

We also believe that based on our findings, clear processes for the effective communication of anticipated and ongoing shortages of medicines, including antimicrobials should be established among all public sector hospitals. These hospitals should design and implement measures to ameliorate weaknesses in the inventory control process as well as minimise disruptions in medicine supply, building on current initiatives in South Africa. These measures should prioritise antimicrobial preservation given concerns with rising AMR rates in South Africa. 

Furthermore, therapeutic interchange policies should be considered for implementation at various levels of care as they take into consideration the spectrum of activity, cost, and associated adverse drug reactions of different antimicrobials, which can be part of AMSPs. This will be followed-up in the future. We hope our findings are of interest to other low to medium income countries experiencing challenges with medicine shortages, especially antimicrobials in hospitals.

## Figures and Tables

**Table 1:** Response rate and study population per province.

Province	Target Population (*n* = 403)	Excluded from Target Population (*n* = 149)				Study Population (*n* = 254)		
		No Approval Response	Approval Denied	Could not be Reached for an Email Address	Declined Participation/no Pharmacist/no Email or Fax	Email Delivered	Non-Response	Responses Per province (%)
Eastern Cape	90	0	0	28	3	59	41	18 (21.2%)
Free State	34	0	1	17	2	14	8	6 (7.1%)
Gauteng	36	26	0	0	0	10	2	8 (9.4%)
Kwa-Zulu Natal	77	0	0	7	0	70	57	13 (15.3%)
Limpopo	40	0	0	4	0	36	28	8 (9.4%)
Mpumalanga	33	0	0	11	1	21	17	4 (4.7%)
Northern Cape	19	0	0	1	3	15	2	13 (15.3%)
North West	20	0	0	1	1	18	10	8 (9.4%)
Western Cape	54	20	10	12	1	11	4	7 (8.2%)
Total number (%)	403	46 (11.4%)	11 (2.7%)	81 (20.1%)	11 (2.7%)	254	169 (66.5%)	85 (33.5%)

**Table 2:** Demographic characteristics of respondents (*n* = 85).

Respondents’ Characteristics		Respondents; n (%)
Gender	Male	31 (36.5%)
	Female	54 (63.5%)
Age (years)	20–30	19 (24.1%)
	˃30–40	28 (35.4%)
	˃40–50	20 (25.3%)
	˃50–65	12 (15.2%)
Years of practice in the public sector	≤10	51 (62.2%)
	˃10–20	24 (29.3%)
	˃20–30	5 (6.1%)
	˃30	2 (2.4%)
Designation	Pharmacy manager	41 (48.2%)
	Pharmacist (completed community service; no specific designation)	19 (22.4%)
	Procurement Pharmacist	13 (15.3%)
	Drug controller	10 (11.8%)
	Clinical Pharmacist	4 (4.7%)
	Community Service Pharmacist	4 (4.7%)
	Production Pharmacist	2 (2.4%)
	Clinical supervisor	1 (1.2%)
	Sub-district Pharmacist	1 (1.2%)
Level of care	District hospital	43 (51.8%)
	Regional hospital	17 (20.5%)
	Tertiary hospital	11 (13.3%)
	Specialised hospital	10 (12.1%)
	Central hospital	3 (3.6%)

**Table 3:** Antimicrobial shortages by Anatomical Therapeutic Chemical classification.

Antimicrobial Class	ATC Classification	Antimicrobial	Number of Respondents ^a^	Total Number (%) Who Reported Antimicrobial as Unavailable	Average Duration of Shortages (days)
Penicillins	J01CF02	Cloxacillin	70	38 (54.3%)	5 to >40
	J01CE08	Benzathine Benzylpenicillin	72	39 (54.2%)	5 to >40
	J01CE02	Phenoxymethylpenicillin IV	58	28 (48.3%)	21 to >40
	J01CE02	Phenoxymethylpenicillin oral	71	31 (43.7%)	5 to >40
	J01CE01	Benzyl Penicillin	72	24 (33.3%)	5 to >40
	J01CE09	Procaine Penicillin	55	14 (25.5%)	21 to >40
	J01CA01	Ampicillin IV	72	15 (20.8%)	5 to >40
B-lactam inhibitor combinations	J01CR02	Amoxicillin/Clavulanic Acid IV	71	21 (29.6%)	5 to >40
	J01CR05	Piperacillin/Tazobactam	61	16 (26.2%)	5 to 40
Macrolides	J01FA01	Erythromycin	53	21 (39.6%)	5 to >40
	J01FA10	Azithromycin IV	59	11 (18.6%)	11 to 40
Cephalosporins	J01DD04	Ceftriaxone	71	27 (38.0%)	5 to >40
	J01DE01	Cefepime	53	14 (26.4%)	5 to >40
Aminoglycosides	J01GB03	Gentamicin	67	19 (28.4%)	5 to >40
Imidazole antifungal	D01AC01	Clotrimazole cream	71	20 (28.2%)	5 to >40
Glycopeptides	J01XA01	Vancomycin Oral	40	11 (27.5%)	>40
	J01XA01	Vancomycin IV	62	13 (21.0%)	11 to 40
Synthetic nucleoside analogue antiviral	D06BB03	Acyclovir IV	58	14 (24.1%)	5 to >40
Tetraene polyene antifungal	D01AA02	Natamycin	43	8 (18.6%)	>40
Polyene antifungal	J02AA01	Amphotericin B	72	13 (18.1%)	5 to >40

^a^ Total number of respondents who indicated the antimicrobial as either available or not available.

**Table 4:** Reasons for, impact of, and procedures for reporting antimicrobial shortages.

Reasons for Antimicrobial Shortages ^a^	Hospital Level of Care					Total (%) (*n* = 75)
	District (*n* = 37)	Regional (*n* = 15)	Tertiary (*n* = 10)	Central (*n* = 3)	Specialised (*n* = 10)	
Pharmaceutical companies with supply or capacity problems	33 (51.6%)	11 (17.2%)	7 (10.9%)	3 (4.7%)	10 (15.6%)	64 (85.3%)
Inefficient supply system from the depot to the facility	31 (56.4%)	10 (11.1%)	8 (14.5%)	1 (1.8%)	5 (9.1%)	55 (73.3%)
Poor stock control systems	9 (50.0%)	5 (27.8%)	2 (11.1%)	0 (0.0%)	2 (11.1%)	18 (24.0%)
Shortage of funds/resources	9 (50.0%)	5 (27.8%)	2 (11.1%)	1 (5.6%)	1 (5.6%)	18 (24.0%)
Wastage of medicines	5 (35.7%)	3 (21.4%)	3 (21.4%)	1 (7.1%)	2 (14.3%)	14 (18.7%)
Increase in the number of patients relying on facility for medication	7 (50.0%)	4 (28.6%)	2 (14.3%)	0 (0.0%)	1 (7.1%)	14 (18.7%)
Lack of reliable information on medicine needs and usage	4 (33.3%)	4 (33.3%)	4 (33.3%)	0 (0.0%)	0 (0.0%)	12 (16.0%)
Unclear lines of accountability	5 (71.4%)	0 (0.0%)	1 (14.3%)	0 (0.0%)	1 (14.3%)	7 (9.3%)
Poor ordering practices by pharmacists or nurses	4 (80.0%)	0 (0.0%)	1 (20.0%)	0 (0.0%)	0 (0.0%)	5 (6.7%)
Protest action resulting in shut down of depot	4 (80.0%)	1 (20.0%)	0 (0.0%)	0 (0.0%)	0 (0.0%)	5 (6.7%)
Lack of storage facilities for medicines	3 (100.0%)	0 (0.0%)	0 (0.0%)	0 (0.0%)	0 (0.0%)	3 (4.0%)
Unapproved tenders/changes to tender	0 (0.0%)	1 (50.0%)	1 (50.0%)	0 (0.0%)	0 (0.0%)	2 (2.7%)
Need to motivate for drugs	0 (0.0%)	1 (100.0%)	0 (0.0%)	0 (0.0%)	0 (0.0%)	1 (1.3%)
Requested quantities too small for depot to supply	0 (0.0%)	0 (0.0%)	0 (0.0%)	0 (0.0%)	1 (100.0%)	1 (1.3%)
Impact of antimicrobial shortages ^a^	District (*n* = 37)	Regional (*n* = 16)	Tertiary (*n* = 11)	Central (*n* = 3)	Specialised (*n* = 10)	Total (%) (*n* = 77)
Dispensed second/third/fourth generation antimicrobials	35 (53.8%)	14 (21.5%)	10 (15.4%)	2 (3.1%)	6 (9.2%)	65 (84.4%)
Dispensed more expensive alternatives	26 (52.0%)	12 (24.0%)	9 (18.0%)	1 (2.0%)	2 (4.0%)	50 (64.9%)
Turned patient away with no medication	14 (63.6%)	3 (13.6%)	2 (9.1%)	1 (4.5%)	2 (9.1%)	22 (28.6%)
Referred patient to a private institution	10 (55.6%)	3 (16.7%)	4 (22.2%)	0 (0.0%)	1 (5.6%)	18 (23.4%)
Procedure for reporting shortages ^a^	District (*n* = 35)	Regional (*n* = 17)	Tertiary (*n* = 8)	Central (*n* = 1)	Specialised (*n* = 7)	Total (%) (*n* = 68)
Weekly reports to central/provincial office or district pharmacist	14 (53.8%)	5 (19.2%)	3 (11.5%)	0 (0.0%)	4 (15.4%)	26 (38.2%)
Report to PTC	6 (40.0%)	4 (26.7%)	3 (20.0%)	0 (0.0%)	2 (13.3%)	15 (22.1%)
Update on inventory management system/out of stock book	8 (61.5%)	4 (30.8%)	1 (7.7%)	0 (0.0%)	0 (0.0%)	13 (19.1%)
Notify prescribers via telephone, email, meetings, SOPs or notices	8 (61.5%)	2 (15.4%)	2 (15.4%)	0 (0.0%)	1 (7.7%)	13 (19.1%)
Notify pharmacy management	4 (33.3%)	4 (33.3%)	3 (25.0%)	1 (8.3%)	0 (0.0%)	12 (17.6%)
Notify the depot	5 (62.5%)	2 (25.0%)	1 (12.5%)	0 (0.0%)	0 (0.0%)	8 (11.8%)
Report to CEO and PTC	3 (42.9%)	1 (14.3%)	1 (14.3%)	0 (0.0%)	2 (28.6%)	7 (10.3%)
Not available	3 (100.0%)	0 (0.0%)	0 (0.0%)	0 (0.0%)	0 (0.0%)	3 (4.4%)

^a^ More than one response option provided. CEO: Chief Executive Officer; PTC: Pharmacy and Therapeutics Committee; SOP: Standard Operating Procedure.

**Table 5:** Therapeutic interchange process by hospital level of care.

Therapeutic interchange Process	Hospital Level of Care					
Therapeutic interchange policy description	District (*n* = 18)	Regional (*n* = 11)	Tertiary (*n* = 5)	Central (*n* = 1)	Specialised (*n* = 4)	Total (%) (*n* = 39)
Memo from National Department of Health	6 (46.2%)	5 (38.5%)	1 (7.7%)	1 (7.7%)	0 (0.0%)	13 (33.3%)
SOP/guidelines from PTCs	7 (77.8%)	2 (22.2%)	0 (0.0%)	0 (0.0%)	0 (0.0%)	9 (23.1%)
Not available	1 (14.3%)	1 (14.3%)	3 (42.9	0 (0.0%)	2 (28.6%)	7 (17.9%)
Hospital notice with alternatives/supplementary list	2 (50.0%)	2 (50.0%)	0 (0.0%)	0 (0.0%)	0 (0.0%)	4 (10.3%)
Internal arrangement between pharmacists and prescribers	1 (25.0%)	1 (25.0%)	0 (0.0%)	0 (0.0%)	2 (50.0%)	4 (10.3%)
Alternatives from the depot	1 (50.0%)	0 (0.0%)	1 (50.0%)	0 (0.0%)	0 (0.0%)	2 (5.1%)
Health personnel responsible for development of therapeutic interchange policies ^a^	District (*n* = 39)	Regional (*n* = 17)	Tertiary (*n* = 11)	Provincial (*n* = 3)	Specialised (*n* = 10)	Total (%) (*n* = 82)
Pharmacy and Therapeutics Committee	25 (61.0%)	6 (14.6%)	3 (7.3%)	3 (7.3%)	4 (9.8%)	41 (50.0%)
Pharmacist	12 (46.2%)	5 (19.2%)	5 (19.2%)	2 (7.7%)	2 (7.7%)	26 (31.7%)
Prescribers	9 (45.0%)	3 (15.0%)	3 (15.0%)	1 (5.0%)	4 (20.0%)	20 (24.4%)
National Department of Health	1 (33.3%)	2 (66.7%)	0 (0.0%)	0 (0.0%)	0 (0.0%)	3 (3.7%)
Microbiologist	0 (0.0%)	2 (100.0%)	0 (0.0%)	0 (0.0%)	0 (0.0%)	2 (2.4%)
Operational manager (registered nurses)	1 (100.0%)	0 (0.0%)	0 (0.0%)	0 (0.0%)	0 (0.0%)	1 (1.2%)
Not applicable	10 (45.5%)	6 (27.3%)	4 (18.2%)	0 (0.0%)	2 (9.1%)	22 (26.8%)
Actions taken by the pharmacist during the therapeutic interchange process ^a^	District (*n* = 41)	Regional (*n* = 17)	Tertiary (*n* = 11)	Provincial (*n* = 3)	Specialised (*n* = 10)	Total (%) (*n* = 82)
Communicate with other health professionals regarding any ongoing medicine shortages and available substitutions	39 (50.6%)	16 (20.8%)	10 (13.0%)	2 (2.6%)	10 (13.0%)	77 (93.9%)
Keep a record of the interchange	25 (49.0%)	10 (19.6%)	8 (15.7%)	2 (3.9%)	6 (11.8%)	51 (62.2%)
Patient counselling	28 (68.3%)	4 (9.8%)	6 (14.6%)	1 (2.4%)	2 (4.8%)	41 (50.0%)
None	2 (100.0%)	0 (0.0%)	0 (0.0%)	0 (0.0%)	0 (0.0%)	2 (2.4%)
Provide prescribers with information on new therapeutic equivalent and correct dosages	1 (100.0%)	0 (0.0%)	0 (0.0%)	0 (0.0%)	0 (0.0%)	1 (1.2%)
Antimicrobial substitution procedures, followed by pharmacists, in the absence of therapeutic interchange policies ^a^	District (*n* = 42)	Regional (*n* = 17)	Tertiary (*n* = 11)	Central (*n* = 2)	Specialised (*n* = 9)	Total (%) (*n* = 81)
Call to notify the prescriber, avail the options and they choose/endorse change before dispensing alternative	38 (52.8%)	14 (19.4%)	10 (13.9%)	3 (4.2%)	7 (9.7%)	72 (88.9%)
Send a written memo to the prescribers supplying the available options	19 (40.4%)	14 (29.8%)	7 (14.9%)	3 (6.4%)	4 (8.5%)	47 (58.0%)
Send the patient back to the prescriber	10 (45.5%)	7 (31.8%)	2 (9.1%)	1 (4.5%)	2 (9.1%)	22 (27.2%)
Consult pharmacists at tertiary level for alternatives	1 (100.0%)	0 (0.0%)	0 (0.0%)	0 (0.0%)	0 (0.0%)	1 (1.2%)
Borrow or buy out from other institutions	1 (100.0%)	0 (0.0%)	0 (0.0%)	0 (0.0%)	0 (0.0%)	1 (1.2%)
Urgent meeting by Rational Medicines Use Committee to discuss way forward	1 (100.0%)	0 (0.0%)	0 (0.0%)	0 (0.0%)	0 (0.0%)	1 (1.2%)

^a^ More than one response option provided.

**Table 6:** Perceived role of the pharmacist in the therapeutic interchange process.

Role of the Pharmacist	Hospital Level of Care					Total (%) (*n* = 70)
	District (*n* = 36)	Regional (*n* = 16)	Tertiary (*n* = 9)	Central (*n* = 1)	Specialised (*n* = 8)	
Share information and communicate with prescribers on rational medicine use and available therapeutic options	12 (46.2%)	6 (23.1%)	4 (15.4%)	1 (3.8%)	3 (11.5%)	26 (37.1%)
Facilitate therapeutic interchange policy development and/or selection of therapeutic alternatives in the PTC	11 (68.8%)	2 (12.5%)	2 (12.5%)	0 (0.0%)	1 (6.3%)	16 (22.9%)
Work as part of a team with clinicians to provide the best therapeutic alternatives for the patient	6 (40.0%)	4 (26.7%)	2 (13.3%)	0 (0.0%)	3 (20.0%)	15 (21.4%)
Participate in research with other health care professionals to make informed medicine choices	2 (33.3%)	2 (33.3%)	1 (16.7%)	0 (0.0%)	1 (16.7%)	6 (8.6%)
Interchange without consulting the prescriber to reduce patient waiting times if all the patient information is available	2 (50.0%)	2 (50.0%)	0 (0.0%)	0 (0.0%)	0 (0.0%)	4 (5.7%)
Educate and counsel patients on changes to their treatment	3 (100.0%)	0 (0.0%)	0 (0.0%)	0 (0.0%)	0 (0.0%)	3 (4.3%)

PTC: Pharmacy and Therapeutics Committee.

## Data Availability

The datasets during and/or analysed during the current study are available from the corresponding author on reasonable request.

## List of Abbreviations

**Abbreviation**	**Meaning**
AMR AMS	Antimicrobial Resistance Antimicrobial Stewardship
AMSCs AMSPs	Antimicrobial Stewardship Committees Antimicrobial Stewardship Programmes
ATC	Anatomical Therapeutic Chemical Classification
CEO	Chief Executive Officer
HIV	Human Immunodeficiency Virus
MS	Microsoft Office
NDoH	National Department of Health
PTC	Pharmacy and Therapeutics Committee
SD	Standard Deviation
SMUREC	Sefako Makgatho University Research Ethics Committee
SOP	Standard Operating Procedures
SPSS	Statistical Package for the Social Sciences
WHO	World Health Organization
